# Preoperative Inflammatory Status and Postoperative Morbidity in Patients With Primary Retroperitoneal Sarcoma

**DOI:** 10.1002/cam4.70588

**Published:** 2025-03-02

**Authors:** Pia van der Laan, Fabio Tirotta, Stijn van der Burg, Stefanie Hakkesteegt, Max L. Almond, Yvonne Schrage, Anant Desai, Winette T. A. van der Graaf, Dirk J. Grunhagen, Samuel J. Ford, Cornelis Verhoef, Winan J. van Houdt

**Affiliations:** ^1^ Department of Surgery, Netherlands Cancer Institute Antoni van Leeuwenhoek Hospital Amsterdam The Netherlands; ^2^ Department of Sarcoma and General Surgery, Midlands Abdominal and Retroperitoneal Sarcoma Unit University Hospitals Birmingham NHS Foundation Trust Birmingham UK; ^3^ Department of Surgical Oncology, Erasmus MC Cancer Institute University Medical Centre Rotterdam Rotterdam The Netherlands; ^4^ Department of Medical Oncology, Netherlands Cancer Institute Antoni van Leeuwenhoek Hospital Amsterdam The Netherlands; ^5^ Department of Medical Oncology, Erasmus MC Cancer Institute University Medical Centre Rotterdam Rotterdam The Netherlands

**Keywords:** C‐reactive protein, liposarcoma, neutrophil‐to‐lymphocyte ratio, postoperative complications, retroperitoneal sarcoma

## Abstract

**Background:**

The role of preoperative inflammatory markers in predicting postoperative outcomes has been investigated in different types of cancer. However, little is known about retroperitoneal sarcoma (RPS). This study aimed to evaluate the association between preoperative inflammatory status and major postoperative morbidity in patients undergoing RPS surgery.

**Methods:**

Data on patients undergoing surgery for primary RPS between 2008 and 2022 at three specialist sarcoma centers were analyzed. The preoperative inflammatory status was evaluated, assessing the C‐reactive protein (CRP) value, the neutrophil/lymphocyte ratio (NLR), and the platelet/lymphocyte ratio (PLR). The primary outcome was 90‐day major postoperative morbidity. The best‐balanced cutoff values to apply in the uni‐ and multivariable analysis were calculated using a receiver operating characteristic (ROC) curve analysis.

**Results:**

Data were available for 239 patients. Major postoperative complications occurred in 52 of 235 patients (22.1%). Increased median values of CRP, NLR, and PLR were significantly higher in patients with dedifferentiated liposarcoma (DDLPS) (*p* < 0.001). As such, further analysis focused only on this specific histotype. On multivariable analysis, after adjusting for potential confounders, the association between increasing CRP and NLR with 90‐day major postoperative morbidity remained significant, with an OR of 2.96 (95% CI: 1.03–8.49, *p* = 0.044) for CRP > 61 mg/L, and with an OR of 4.69 (1.55–14.20, *p* = 0.006) for NLR > 4.85.

**Conclusion:**

Elevated preoperative levels of CRP and NLR are independently associated with major postoperative morbidity in patients affected by primary retroperitoneal DDLPS. These findings may help decision‐making and optimize perioperative management in these patients.

## 1 Introduction

Retroperitoneal sarcoma (RPS) is a rare group of different diseases, which require a bespoke surgical approach depending on the histology [1]. RPS surgery is complex and characterized by approximately 20% rate of major postoperative morbidity [2]. In the past decades, there has been an overall improvement in surgical care, in both surgical techniques and patient perioperative management, which has been translated into a reduction of postoperative mortality in most surgical procedures. However, postoperative morbidity in major abdominal surgery, such as RPS surgery, is still common and has a relevant impact on patients' quality of life and hospital costs [3]. Trying to mitigate the postoperative complication burden, identifying high‐risk patients has therefore become crucial. With this in mind, several studies in different types of cancer analyzed the role of the preoperative inflammatory status, aiming to identify its association with postoperative morbidity [4, 5, 6]. Particular attention has been given to some easily available and routinely performed markers such a C‐reactive protein (CRP) or the neutrophil‐to‐lymphocyte ratio (NLR). Elevated serum levels of both markers represent the result of the systemic inflammatory response induced by the local immune system related to the presence of a tumor [7]. Hypothetically, this may result in a catabolic status which may lead to an increased risk of developing postoperative complications. There is, however, a lack of evidence about the role of the preoperative inflammatory status in patients with primary RPS. Therefore, the aim of this study was to evaluate the association between preoperative inflammatory status and major postoperative morbidity in patients undergoing surgery for primary RPS with focus on patients with dedifferentiated liposarcoma (DDLPS).

## 2 Methods

### 2.1 Data Collection

Data on patients undergoing surgery for primary RPS between January 1, 2008, and December 31, 2022, were extracted from three specialist sarcoma centers' databases:

1. University Hospitals Birmingham NHS Foundation Trust, Birmingham, United Kingdom
2. The Netherlands Cancer Institute – Antoni van Leeuwenhoek, Amsterdam, the Netherlands
3. Erasmus MC Cancer Institute, University Medical Centre Rotterdam, Rotterdam, the Netherlands

Exclusion criteria were the presence of metastatic or recurrent disease at the time of surgery or patients who did not have a preoperative CRP available 60 days before surgery.

Ethical approval was obtained from the Institutional Review Board of The Netherlands Cancer Institute (IRBd2 3‐299). This retrospective cohort study was conducted using the “Strengthening the report of observational studies in epidemiology (STROBE)” guidelines [8].

Data were available for age, sex, and body mass index (BMI). Preoperative values of CRP, albumin, neutrophils, lymphocytes, and platelet counts 60 days before surgery were also available. In addition, tumor‐related data including tumor histology, location, grade, size, and multifocality, as well as treatment‐related factors such as the number of organs resected, resection margin status, and the use of chemo‐ and radiotherapy were considered. Frailty and comorbidities were quantified by the Eastern Cooperative Oncology Group (ECOG) performance status. Tumor histology was classified as follows: well‐differentiated liposarcoma (WDLPS), DDLPS, leiomyosarcoma (LMS), or “other.” Tumor grading used the French *Fédération Nationale des Centres de Lutte Contre le Cancer* (FNCLCC) grading system [9]. Tumor grading was not applicable for all tumor histologies (e.g., PEComa), or for patients who had received preoperative chemo‐ or radiotherapy.

The organ resected weighted score (ORWS), which assigns a different weight to each organ excised during surgery, was used to quantify the extent of surgery [10].

### 2.2 Preoperative Inflammatory Status

The preoperative inflammatory status was evaluated by assessing the CRP values of every patient operated on a maximum of 60 days before their surgery. Using the same threshold, other surrogate indexes of preoperative inflammatory status were evaluated, such as the CRP/albumin ratio (CAR), the neutrophil/lymphocyte ratio (NLR), and the platelet/lymphocyte ratio (PLR).

### 2.3 Outcomes

The primary outcome was 90‐day major postoperative morbidity. Postoperative complications were quantified based on the Clavien–Dindo Classification (CDC) and major postoperative complications were classified accordingly if patients developed at least one complication graded ≥ 3 [11]. If more than one major postoperative complication had occurred, only the highest complication grade was considered.

### 2.4 Statistical Methods

Data are presented using frequency (%), medians (interquartile range [IQR]), or mean (standard deviation) for continuous variables. Associations between the inflammatory markers and clinicopathological characteristics were studied using Mann–Whitney *U*‐tests or Kruskal–Wallis tests, where appropriate. Correlations between the inflammatory markers were evaluated using Spearman's rank correlation. Associations between inflammatory markers and 90‐day major postoperative complications were then assessed using binary logistic regression models. Initially, univariable models were produced for each of the inflammatory markers, as well as patient and operative factors. A receiver operating characteristic (ROC) curve was used to determine the optimal cutoff for CRP, NLR, and PLR. The most optimal cutoff value was calculated using the Youden Index, which was used to dichotomize these variables. The linearity assumption of continuous variables was assessed using the Box–Tidwell transformation. Independent variables were tested for multicollinearity using the variance inflation factor, and showed multicollinearity for CRP and CAR. Multivariable models were then produced, to assess whether the inflammatory markers were independently associated with 90‐day major complications. Based on the number of events, three variables with the lowest *p*‐value in the univariate models were entered into the multivariable models alongside the inflammatory markers. Separate models were produced for each inflammatory marker, due to the high level of correlation between the markers. *p* < 0.05 was considered as statistically significant. Statistical analyses were performed using IBM SPSS statistical software version 29.0.

## 3 Results

### 3.1 Cohort Characteristics

In the study period, data were available for 239 patients. The mean age at diagnosis was 60.2 ± 13.7 years. The majority of patients were male (133 of 239 [55.6%]). DDLPS was the most common histology (105 of 239 [43.19%]) followed by “other” (49 of 239 [20.5%]), LMS (46 of 239 [19.2%]), and WDLPS (39 of 239 [16.3%]). The median tumor size was 200 mm (IQR: 130–300).

Median values for preoperative inflammatory markers, namely CRP, CAR, NLR, and PLR, were 10 mg/L (IQR: 3–51), 0.28 (IQR: 0.08–1.41), 3.16 (IQR: 1.76–5.53), and 173.33 (IQR: 91.9–293.22), respectively. Major postoperative complications occurred in 52 of 235 patients (22.1%) including 2 patients who died of postoperative complications within 90 days from surgery. Further details are summarized in Table 1.

**TABLE 1:** Patient tumor and treatment characteristics.

Factor	Whole cohort	
	*n*	Statistic
Age at diagnosis, years (mean)	239	60.2 ± 13.7
Sex (% male)	239	133 (55.6)
ECOG performance status	206	
0		144 (60.3)
1		48 (20.1)
2–3		14 (5.9)
Tumor histology	239	
DDLPS		105 (43.9)
WDLPS		39 (16.3)
LMS		46 (19.2)
Other		49 (20.5)
Tumor location	239	
Right retroperitoneum		99 (41.6)
Left retroperitoneum		89 (37.4)
Other		50 (20.9)
Tumor largest dimension (mm)	239	200 (130–300)
FNCLCC tumor grade	206^a^	
1		55 (26.7)
2		51 (24.8)
3		100 (48.5)
Multifocal	239	8 (3.3)
Organ resected weighted score	239	
0–3		205 (85.8)
≥ 4		34 (14.2)
Resection margins, % R2	239	11 (4.6)
Chemotherapy	239	
No		224 (93.7)
Preoperative		15 (6.3)
Radiotherapy	239	
No		219 (91.6)
Preoperative		15 (6.3)
Postoperative		5 (2.1)
Preoperative CRP (mg/L)	239	10 (3‐51)
Preoperative CAR	232	0.28 (0.08–1.41)
Preoperative NLR	226	3.16 (1.76–5.53)
Preoperative PLR	230	173.33 (91. 9‐293.22)
Major 90‐day postoperative complications^b^	235	52 (22.1)

*Note:* Data are reported as *n* (%), median (interquartile range) or mean ± standard deviation.

Abbreviations: CAR, C‐reactive protein/albumin ratio; CRP, C‐reactive protein; DDLPS, Dedifferentiated liposarcoma; ECOG, Eastern Cooperative Oncology Group; FNCLCC, Fédération Nationale des Centres de Lutte Contre Le Cancer; LMS, Leiomyosarcoma; NLR, neutrophil–lymphocyte ratio; PLR, platelet–lymphocyte ratio; WDLPS, well‐differentiated liposarcoma.

^a^ Tumor grading was not applicable for all tumor histologies (e.g., PEComa), or for patients who had received preoperative chemo‐ or radiotherapy.

^b^ Patients with histologies Clavien–Dindo Classification grade ≥ 3.

### 3.2 Correlation Between Inflammatory Markers

The correlation between inflammatory markers was evaluated and is summarized in Table 2. A near‐perfect correlation between CRP and CAR (*r* = 0.99) was observed. As such, CAR was excluded from subsequent analyses.

**TABLE 2:** Correlation between inflammatory markers.

Variables		CRP	CAR	NLR	PLR
CRP (mg/L)	Correlation coefficient	—	0.993	0.432	0.439
	*p*‐value	—	**< 0.001**	**< 0.001**	**< 0.001**
	*n*	—	232	226	230
CAR	Correlation coefficient	0.993	—	0.455	0.469
	*p*‐value	**< 0.001**	—	**< 0.001**	**< 0.001**
	*n*	232	—	224	227
NLR	Correlation coefficient	0.432	0.455	—	0.851
	*p*‐value	**< 0.001**	**< 0.001**	**—**	**< 0.001**
	*n*	226	224	—	226
PLR	Correlation coefficient	0.439	0.469	0.851	—
	*p*‐value	**< 0.001**	**< 0.001**	**< 0.001**	—
	*n*	230	227	226	—

Abbreviations: CAR, C‐reactive protein–albumin ratio; CRP, C‐reactive protein; NLR, neutrophil–lymphocyte ratio; PLR, platelet–lymphocyte ratio. Bold *p*‐values are significant at *p* < 0.05.

### 3.3 Associations With Preoperative Inflammatory Markers

The median values of CRP, NLR, and PLR were used to evaluate their association with clinicopathological factors.

Increased CRP was significantly higher in patients with ECOG ≥ 1 (*p* = 0.006), DDLPS on histology (*p* < 0.001), tumors located in the left retroperitoneum (*p* < 0.022), large tumor size (*p* < 0.001), grade 3 tumors (*p* < 0.001), ORWS ≥ 4 (*p* < 0.001), in patients who did not receive preoperative chemotherapy (*p* = 0.004), and in those who developed major postoperative morbidity (*p* = 0.002).

Increased NLR was significantly associated with DDLPS on histology (*p* < 0.001), larger tumor size (*p* < 0.001), left‐sided tumors (*p* < 0.017), grade 3 tumors (*p* < 0.001), ORWS ≥ 4 (*p* < 0.001), in patients who did not receive preoperative chemotherapy (*p* = 0.003), and in those who developed major postoperative morbidity (*p* < 0.001). Further details are summarized in Table 3.

**TABLE 3:** Associations of clinicopathological factors with inflammation markers.

Factor	CRP (mg/L)			NLR			PLR		
	Median [IQR]	*n*	*p*	Median [IQR]	*n*	*p*	Median [IQR]	*n*	*p*
Age at diagnosis (years)			0.235			0.191			0.519
< 60	7.0 [3.0–35.3]	102		3.0 [1. 6‐5.0]	93		162.1 [88. 2‐284.9]	97	
≥ 60	14.0 [3.0–58.0]	137		3.3 [1. 8‐6.0]	133		180.0 [95. 7‐303.2]	133	
Sex			0.753			0.044			0.395
Male	14.0 [3.0–56.5]	133		3.5 [1. 9‐7.0]	125		182.7 [94. 7‐300.4]	126	
Female	9.0 [3.0–40.0]	106		2.7 [1. 6‐4.8]	101		159.5 [88.0–277.0]	104	
ECOG performance status			**0.006**			0.018			**0.008**
0	7.0 [3.0–31.8]	144		3.3 [2. 1‐5.3]	135		180.0 [114. 5‐278.3]	136	
≥ 1	23.5 [4.0–122.5]	62		4.5 [2. 5‐7.5]	61		228.9 [135. 4‐3760.4]	61	
Tumor histology			**< 0.001**			**< 0.001**			**< 0.001**
DDLPS	31.0 [7.0–101.0]	105		4.2 [2. 7‐7.0]	97		222.8 [143. 5‐364.0]	101	
WDLPS	3.0 [2.0–9.0]	39		2.7 [1. 5‐6.2]	38		148.0 [89. 3‐307.4]	38	
LMS	5.0 [3.0–29.0]	46		2.1 [0. 7‐4.1]	46		124.4 [52. 9‐229.0]	46	
Other	5.0 [2.0–31.0]	49		2.4 [1. 6‐4.5]	45		153.9 [91. 4‐221.5]	45	
Tumor location			**0.001**			**0.017**			**0.008**
Right retroperitoneum	9.0 [3.0–31.0]	99		3.2 [1. 8‐5.9]	93		173.6 [97. 2‐283.6]	94	
Left retroperitoneum	24.0 [3.0–102.0]	89		3.5 [2. 1‐6.4]	84		209.4 [104. 4‐374.2]	86	
Other	4.5 [2.0–31.0]	50		2.3 [1. 1‐4.5]	48		134.2 [62. 8‐209.1]	49	
Tumor largest dimension (per 10 cm)			**< 0.001**			**< 0.001**			**< 0.001**
< 2	4.0 [3.0–23.5]	108		2.3 [0. 9‐4.3]	101		138.0 [54. 5‐224.5]	103	
≥ 2	23.0 [4.0–80.0]	131		3.92 [2. 3‐6.3]	125		214.0 [135. 4‐356.9]	127	
FNCLCC tumor grade			**< 0.001**			**0.001**			**< 0.001**
1	3.0 [2.0–9.0]	55		2.3 [1. 6‐4.1]	54		146.7 [85. 1‐191.9]	54	
2	10.0 [3.0–33.0]	51		3.1 [1. 8‐5.6]	51		181.3 [101. 4‐280.8]	51	
3	38.0 [7. 5‐129.5]	100		4.4 [2. 6‐7.1]	96		248.7 [143. 9‐383.3]	97	
Multifocal			0.243			0.547			0.405
No	10.0 [3.0–51.0]	231		3.2 [1. 8‐5.4]	218		170.9 [90. 2‐293.2]	221	
Yes	4.0 [1. 3‐53.8]	8		3.6 [1. 8‐12.6]	8		180.3 [149. 5‐367.9]	9	
Organ resected weighted score			**< 0.001**			**0.002**			**< 0.001**
0–3	7.0 [3.0–32.5]	205		3.0 [1. 6‐5.3]	193		159.6 [83. 3‐263.2]	196	
≥ 4	49.0 [25. 3‐141.0]	34		4.8 [2. 7‐7.7]	33		287.8 [180. 5‐471.1]	34	
Resection margins			0.440			0.901			0.255
R0–R1	10.0 [3.0–49.8]	228		3.2 [1. 7‐5.7]	218		174.7 [93.0–295.3]	221	
R2	27.0 [3.0–69.0]	11		2.9 [2. 3‐5.1]	8		127.1 [46. 1‐247.0]	9	
Chemotherapy			**0.002**			**0.003**			0.069
No	11.5 [3.0–53.5]	224		3.3 [1. 8‐5.6]	211		174.7 [95.0–298.5]	215	
Preoperative	3.0 [2.0–5.0]	15		1.5 [0. 6‐3.2]	15		135.0 [43. 9‐228.9]	15	
Radiotherapy			0.094			0.969			0.761
No	10.0 [3.0–52.0]	219		3.1 [1. 8‐5.5]	207		170.1 [91. 9‐291.1]	210	
Preoperative	5.0 [3.0–19.0]	15		4.4 [1. 4‐5.4]	14		180.0 [124. 5‐295.6]	15	
Postoperative	51.0 [18.0–282.0]	5		3.3 [1. 1‐16.7]	5		398.0 [37.0–701.7]	5	
90‐day postoperative complications			**0.002**			**< 0.001**			**< 0.001**
Clavien–Dindo grade < 3	7.0 [3.0–39.0]	183		3.0 [1. 5‐5.0]	172		158.7 [82. 7‐263.8]	174	
Clavien–Dindo grade ≥ 3	30.0 [5.0–125.5]	52		5.0 [2. 2‐8.3]	50		220.4 [151. 5‐383.1]	52	

*Note:* Data are reported as median (interquartile range), with *p*‐values from Mann–Whitney *U*‐tests or Kruskal–Wallis tests. Bold *p*‐values are significant at *p* < 0.05.

Abbreviations: CRP, C‐reactive protein; DDLPS, dedifferentiated liposarcoma; ECOG, Eastern Cooperative Oncology Group; FNCLCC, Fédération Nationale des Centres de Lutte Contre Le Cancer; LMS, Leiomyosarcoma; NLR, neutrophil–lymphocyte ratio; PLR, platelet–lymphocyte ratio; WDLPS, well‐differentiated liposarcoma.

**FIGURE 1:**
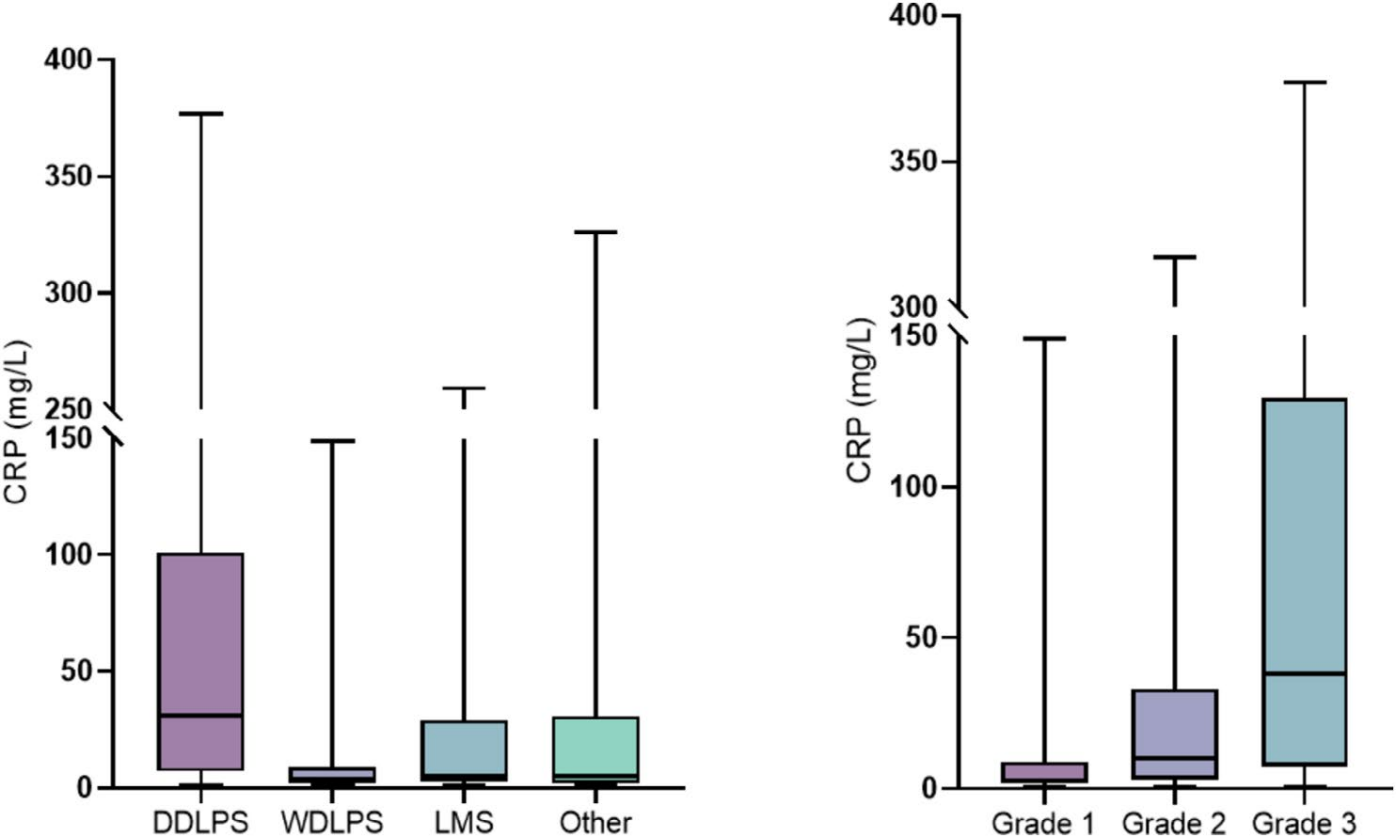
Median preoperative CRP values for tumor histology and grade. CRP, C‐reactive protein; DDLPS, dedifferentiated liposarcoma; LMS, leiomyosarcoma; WDLPS, well‐differentiated liposarcoma.

Because the median inflammatory marker values were significantly higher in patients with DDLPS compared to other histologies, the subsequent analysis focused only on this specific subtype. A graphic illustration of the association between CRP and tumor histology and grade is shown in Figure 1.

### 3.4 ROC Analysis

ROC curves were then produced to assess the associations between the systemic inflammatory markers and major postoperative complications in patients with DDLPS. CRP was found to be the strongest predictor of this outcome, with an AUC of 0.70 (95% CI: 0.59–0.81, *p* = 0.002), which was followed by NLR (AUC 0.65, 95% CI: 0.52–0.77, *p* = 0.028); PLR was not found to be significantly predictive of major postoperative complications in this analysis (AUC 0.59, 95% CI, 0.46–0.72, *p* = 0.156). The ROC curves for each marker were then further interrogated to identify the optimum cutoff value for the prediction of major postoperative complications. This identified values of 61.0 mg/L, 4.85, and 345.2 for CRP, NLR, and PLR, respectively, with values above these thresholds classified as “elevated” for subsequent analysis.

### 3.5 Univariable Analysis for DDLPS

On univariable analysis, patients with a CRP > 61.0 mg/L were found to have a significantly higher rate of major postoperative complications, yielding an OR of 4.30 (1.72–10.74, *p* = 0.002). Patients with a NLR > 4.85 also showed a significantly higher rate of major postoperative complications, with an OR of 2.86 (1.49–9.98, *p* = 0.005). This finding was also consistent in patients with a PLR > 345.2, reporting an OR of 2.80 (1.12–7.04, *p* = 0.028). Further details are shown in Table 4.

**TABLE 4:** Univariable analysis of 90‐day major postoperative complications for dedifferentiated liposarcoma.

	Univariable	
	OR (95% CI)	*p*
Age at diagnosis (per decade)	1.47 (0.98–2.22)	0.060
Sex (% male)		
Male	Ref	
Female	0.98 (0.41–2.35)	0.959
ECOG performance status		
0	Ref	
≥ 1	2.81 (1.09–7.28)	**0.033**
Tumor location		0.396
Right retroperitoneum	Ref	
Left retroperitoneum	1.80 (0.72–4.53)	0.212
Other	0.90 (0. 16‐4.93)	0.903
Tumor largest dimension (per 10 cm)	1.06 (0.74–1.51)	0.761
FNCLCC tumor grade		0.781
1	Ref	
2	2.00 (0. 19‐21.43)	0.567
3	1.47 (0. 15‐14.04)	0.738
Multifocal	NA	NA
Organ resected weighted score		
0–3	Ref	
≥ 4	5.12 (1.92–13.64)	**0.001**
Resection margins		
R0–R1	Ref	
R2	2.85 (0.38–21.25)	0.308
Chemotherapy		
No	Ref	
Preoperative	NA	NA
Radiotherapy		
No	Ref	
Preoperative	5.62 (0.49–64.54)	0.166
Inflammatory markers		
CRP (> 61 mg/L)	4.30 (1.72–10.74)	**0.002**
NLR (> 4.85)	3.86 (1.49–9.98)	**0.005**
PLR (> 345.2)	2.80 (1. 12‐7.04)	**0.028**

*Note:* Univariable analyses are from logistic regression models. Bold *p*‐values are significant at *p* < 0.05.

Abbreviations: CRP, C‐reactive protein; ECOG, Eastern Cooperative Oncology Group; FNCLCC, Fédération Nationale des Centres de Lutte Contre Le Cancer; NLR, neutrophil–lymphocyte ratio; PLR, platelet–lymphocyte ratio.

### 3.6 Multivariable Analysis for DDLPS

A multivariable analysis was then performed to assess whether elevated inflammatory markers were independently predictive of patient outcomes, after adjusting for other potentially confounding factors. Analysis of 90‐day major postoperative complications found increasing CRP, NLR, and ORWS to be associated with a significantly higher rate of postoperative complications. After adjusting for these factors, the association between increasing CRP and NLR with major postoperative morbidity remained significant, with an OR of 2.96 (95% CI: 1.03–8.49, *p* = 0.044) for CRP > 61 mg/L, and with an OR of 4.69 (1.55–14.20, *p* = 0.006) for NLR > 4.85. Further details are summarized in Table 5.

**TABLE 5:** Multivariable analysis of 90‐day major postoperative complications for dedifferentiated liposarcoma.

	CRP (> 61 mg/L)		NLR (> 4.85)		PLR (> 345.2)	
	OR (95% CI)	*p*	OR (95% CI)	*p*	OR (95% CI)	*p*
Elevated inflammatory markers	2.96 (1.03–8.49)	**0.044**	4.69 (1.55–14.20)	**0.006**	2.22 (0.77–6.45)	0.142
ECOG performance status						
0	Ref		Ref		Ref	
≥ 1	2.28 (0.80–6.53)	0.124	2.51 (0.86–7.34)	0.092	2.29 (0.81–6.49)	0.118
Organ resected weighted score						
0–3	Ref		Ref		Ref	
≥ 4	5.10 (1.73–15.01)	**0.003**	5.26 (1.70–16.24)	**0.004**	4.13 (1.38–12.36)	**0.011**

*Note:* Multivariable analyses are from logistic regression models. Bold *p*‐values are significant at *p* < 0.05.

Abbreviations: CRP, C‐reactive protein; NLR, neutrophil–lymphocyte ratio; PLR, platelet–lymphocyte ratio.

## 4 Discussion

Our study demonstrated that an elevated preoperative inflammatory status is significantly associated with major postoperative morbidity in patients with primary retroperitoneal DDLPS. On univariable analysis, increased CRP, NLR, and PLR were significantly associated with 90‐day major postoperative complications. However, after adjusting for potential confounders, only CRP > 61 mg/L and NLR > 4.85 remained associated with major postoperative morbidity.

The importance of inflammation in cancer development is well known. Several mediators contribute to the complex interaction between tumor–host immune and inflammatory response [12]. Among others [13], both preoperative levels of neutrophils, in the form of NLR, and CRP have been investigated as prognostic factors in several types of cancer including soft tissue sarcoma [13, 14, 15, 16, 17]. However, only a couple of these studies [14,15] evaluated as a secondary outcome whether an elevated preoperative inflammatory status is associated with a higher postoperative complication rate in patients with primary RPS. Netanyahu et al. did not find any significant association between increased NLR or CRP with postoperative complications. Nevertheless, the total number of patients included in the study was low and only 27 patients had a preoperative CRP value available. Likewise, Fiore et al., analyzing a large group of patients with primary RPS, did not find NLR being significantly associated with 30‐day postoperative complications. However, when the preoperative inflammation status was evaluated creating a prognostic score, a significant association between the latter and 30‐day postoperative infective complications was found. In contrast with our study, other histologies aside from DDLPS were considered, and a shorter postoperative cutoff period (30‐day) was applied. In addition, CRP was not evaluated. A different study from *Institut Curie* in Paris, France, described the role of preoperative CRP in the context of the Prognostic Inflammatory and Nutritional Index (PINI) and postoperative complications in patients with retroperitoneal DDLPS [18]. This score, obtained by evaluating both albumin and CRP, showed that patients with an increased PINI experienced more postoperative complications and a longer hospital stay. Despite its descriptive nature and that no adjustments with potential confounders on the uni‐ or multivariable analyses were performed, this study highlighted the relationship between preoperative inflammation in this subgroup of patients and the consequences that this may have on short‐term postoperative outcomes.

Patients with DDLPS usually present with very large and necrotic tumors, and their increased systemic inflammatory status could be a reflection of their relatively advanced disease. Both albumin and CRP are proteins synthesized by the liver in response to pro‐inflammatory cytokines such as interleukin‐6 [19]. The systemic inflammatory status caused by cancer can decrease the concentration of albumin by increasing transcapillary escape. This catabolic cancer‐induced status is likely to cause malnutrition, affecting healing processes necessary after a complex procedure such as RPS surgery, and this may explain the higher likelihood of developing major postoperative complications in these patients.

Our study demonstrated that patients with DDLPS with increased CRP and NLR have a higher risk of developing major postoperative complications and could be easily spotted with a simple and routinely available blood test. The importance of recognizing patients at high risk of major postoperative complications may have important implications. Identifying patients with elevated preoperative CRP and NLR could facilitate the decision‐making process about the type of surgery to be performed. For example, a defunctioning ileostomy may be considered in frail patients who require a compartmental resection. Moreover, patients with increased CRP and/or NLR could be considered for prehabilitation programs, especially in the context of elderly patients, as demonstrated by their carrying a higher risk of postoperative morbidity and mortality compared with non‐elderly patients [20].

The current study has several strengths, including the advantage of evaluating common markers such as CRP and NLR to assess the preoperative inflammatory status. Moreover, in comparison with other studies, we focused on a relatively homogenous group of patients with primary retroperitoneal DDLPS operated on in three European Institutions. Nevertheless, some important limitations must be acknowledged. Firstly, the retrospective nature of the study. Secondly, not every patient undergoing surgery for primary RPS had a preoperative CRP value available, which could have determined a selection bias; the reason why some patients did not have CRP available is unknown, but it is possible that this was due to a random selection of blood tests to undertake before surgery and that in some cases, given the lack of clinical relevance so far, CRP was not considered. Thirdly, although the multivariable analysis adjusted for several potentially relevant confounders, there are likely other unmeasured variables, which may have determined a degree of residual confounding.

In conclusion, our study demonstrated that patients affected by primary retroperitoneal DDLPS with an increased inflammatory status have a higher risk of developing major postoperative complications. Postoperative morbidity in RPS surgery is high, and recognizing and possibly preventing postoperative complications should be considered by every surgical oncologist dealing with this rare and complex disease. In view of our findings, the routine evaluation of CRP and NLR in RPS patients before surgery may be beneficial to optimize perioperative management as well as the surgical strategy. Future studies with longer follow‐up will be able to determine whether CRP is associated with long‐term prognosis as well, and potentially to incorporate it in prognostic tools.

## Author Contributions

**Pia van der Laan:** conceptualization (equal), data curation (equal), formal analysis (lead), methodology (equal), writing – review and editing (equal). **Fabio Tirotta:** conceptualization (lead), data curation (lead), formal analysis (equal), investigation (lead), methodology (lead), project administration (lead), resources (equal), validation (equal), visualization (lead), writing – original draft (lead), writing – review and editing (lead). **Stijn van der Burg:** data curation (equal), formal analysis (equal), writing – review and editing (equal). **Stefanie Hakkesteegt:** data curation (equal), writing – review and editing (equal). **Max L. Almond:** writing – review and editing (equal). **Yvonne Schrage:** writing – review and editing (equal). **Anant Desai:** writing – review and editing (equal). **Winette T. A. van der Graaf:** writing – review and editing (equal). **Dirk J. Grunhagen:** writing – review and editing (equal). **Samuel J. Ford:** writing – review and editing (equal). **Cornelis Verhoef:** writing – review and editing (equal). **Winan J. van Houdt:** data curation (equal), writing – review and editing (equal).

## Ethics Statement

Ethical approval was obtained from the Institutional Review Board of The Netherlands Cancer Institute (IRBd23‐299). Given the retrospective nature of the study, it was not possible to obtain written informed consent from the patients, and a waiver was granted by the local IRB for this purpose.

## Conflicts of Interest

The authors declare no conflicts of interest.

## Data Availability

The data that support the findings of this study are available from the corresponding author upon reasonable request.
